# Royal College of Psychiatrists Paediatric Liaison Network: Training Initiative, 2021–2024

**DOI:** 10.1192/bjo.2024.323

**Published:** 2024-08-01

**Authors:** Ashy Rengit

**Affiliations:** Paediatric Liaison Network, England, United Kingdom

## Abstract

**Aims:**

The primary objective of this project was to gather information from psychiatric trainees across the UK regarding paediatric liaison psychiatry training. It was envisioned that a national survey would yield important information regarding trainee awareness of this sub-specialty, and their training experiences.

The secondary objective of this project was to build an online resource, that could practically address unmet training needs in this area.

**Methods:**

A national survey was undertaken between 29th November 2021 and 17th January 2022, with trainees from RCPsych Liaison Psychiatry, and Child & Adolescent Psychiatry faculties, invited to participate.

Following this survey, the predominant themes in trainee feedback informed the design of an online resource for psychiatry trainees interested in paediatric liaison psychiatry.

**Results:**

Overall, 40 trainees across the UK completed the survey, detailing their views and experiences of paediatric liaison psychiatry training.

While 65% of trainees were aware of paediatric liaison psychiatry as a field, only 37.5% had exposure to the specialty. Approximately 48% of respondents were in training programs that included paediatric liaison psychiatry placements.

Common challenges in accessing paediatric liaison training included; lack of qualified trainers, limited paediatric liaison psychiatry services locally, and competing training commitments. Trainees highlighted the need for a wider network to raise awareness of this sub-specialty, and advertise formal clinical/ research opportunities.

Following this survey, an online resource was constructed with input from the wider Paediatric Liaison Network (PLN) membership, including the following features –

- Clinical opportunities – Paediatric liaison psychiatry department contacts for trainees to access training opportunities.
- Research & Education – Resources useful for trainees interested in paediatric liaison psychiatry.
- Careers – Articles on different career pathways in paediatric liaison psychiatry.

**Conclusion:**

Trainees predominantly reported systemic issues with accessing experience in paediatric liaison psychiatry, especially regarding training program structure and local clinical systems. It is likely that time, and the involvement of health and training providers, is required to address these issues.

However, it is hoped that building this digitally accessible initiative is a step forward in raising awareness, and supporting trainees in building positive experiences within paediatric liaison psychiatry.

Many thanks to all the psychiatry trainees, and members of the Paediatric Liaison Network, who supported this digital project with their feedback. For further details on this initiative, please visit – https://sites.google.com/view/plntrainees/pln-home.

